# Whole-Genome Sequencing and Comparative Genomics of Three *Helicobacter pylori* Strains Isolated from the Stomach of a Patient with Adenocarcinoma

**DOI:** 10.3390/pathogens10030331

**Published:** 2021-03-12

**Authors:** Montserrat Palau, Núria Piqué, M. José Ramírez-Lázaro, Sergio Lario, Xavier Calvet, David Miñana-Galbis

**Affiliations:** 1 Secció de Microbiologia, Departament de Biologia, Sanitat i Medi Ambient, Facultat de Farmàcia i Ciències de l’Alimentació, Universitat de Barcelona, Av. Joan XXIII 27-31, 08028 Barcelona, Catalonia, Spain; montsepalau.8@gmail.com (M.P.); npique@ub.edu (N.P.); 2 Digestive Diseases Service, Hospital de Sabadell, Institut Universitari Parc Taulí-UAB, Parc Tauli 1, 08208 Sabadell, Catalonia, Spain; MRamirezL@tauli.cat (M.J.R.-L.); slario@outlook.es (S.L.); xcalvet@tauli.cat (X.C.); 3 Centro de Investigación Biomédica en Red de Enfermedades Hepáticas y Digestivas (CIBERehd), Instituto de Salud Carlos III, Monforte de Lemos 3–5, 28029 Madrid, Community of Madrid, Spain

**Keywords:** *Helicobacter pylori*, genomic comparison, virulence factors, gastric adenocarcinoma

## Abstract

*Helicobacter pylori* is a common pathogen associated with several severe digestive diseases. Although multiple virulence factors have been described, it is still unclear the role of virulence factors on *H. pylori* pathogenesis and disease progression. Whole genome sequencing could help to find genetic markers of virulence strains. In this work, we analyzed three complete genomes from isolates obtained at the same point in time from a stomach of a patient with adenocarcinoma, using multiple available bioinformatics tools. The genome analysis of the strains B508A-S1, B508A-T2A and B508A-T4 revealed that they were *cagA*, *babA* and *sabB/hopO* negative. The differences among the three genomes were mainly related to outer membrane proteins, methylases, restriction modification systems and flagellar biosynthesis proteins. The strain B508A-T2A was the only one presenting the genotype *vacA s1*, and had the most distinct genome as it exhibited fewer shared genes, higher number of unique genes, and more polymorphisms were found in this genome. With all the accumulated information, no significant differences were found among the isolates regarding virulence and origin of the isolates. Nevertheless, some B508A-T2A genome characteristics could be linked to the pathogenicity of *H. pylori*.

## 1. Introduction

*Helicobacter pylori* is a Gram-negative bacterium that persistently infects the human stomach inducing chronic inflammation. The pathogenicity of *H. pylori* ranges from asymptomatic colonization to bacterially mediated oncogenesis [1]. This bacterium can cause several gastrointestinal diseases, such as gastritis, peptic ulcer disease, gastric adenocarcinoma, and mucosa-associated lymphoid tissue (MALT) lymphoma.

The genomes of *H. pylori* are highly variable, with considerable allelic diversity. This genomic plasticity is thought to aid in its adaptation, which is essential for its survival in different human populations [2]. It has been reported that isolates associated with different geographic areas, different diseases and different individuals might have variable genomic features [3,4,5]. Several genes, such as *vacA*, *cagA*, and *iceA*, among others, have been identified as markers of enhanced pathogenicity of *H. pylori* [6].

All *H. pylori* strains carry the vacuolating cytotoxin gene (*vacA*). This gene contains three highly variable polymorphic regions, which are the signal sequence region (*s1*, *s2*), the intermediate region (*i1*, *i2*, *i3*) and the mid region (*m1*, *m2*). Cytotoxicity is related to variations in the signal and mid regions. Strains of *H. pylori* with the *s1/m1* genotype are more frequently associated with severe disease symptoms than strains carrying other combinations of these alleles [7]. Specifically, *s* region variations are associated with the vacuolating activity of the protein VacA, whereas variations in the *m* region determine the cell specificity of vaculation, by affecting the binding of the toxin to the host cells [8]. Toxin production is highest in *s1/m1* strains, moderate in *s1/m2* strains, and scarce or null in *s2/m2* strains [9]. The *i1* variants of VacA have been shown to have a stronger vacuolating activity than toxins containing the *i2* regions [10].

The *cagA* gene is a marker for the presence of the *cag* pathogenicity island (*cag*-PAI), of approximately 40 kb, whose presence is associated with more severe clinical outcomes. A type IV secretion system translocates CagA protein into gastric epithelial cells, where it is phosphorylated. When this modification occurs, CagA affects various cellular processes and signal transduction pathways, such as disruption of tight and adherent junctions that lead to proinflammatory and mitogenic responses–effects [6]. Studies on *H. pylori* heterogeneity have proved that the strongest virulence factors were among the genes within the *cag*-PAI.

The ‘induced by contact with epithelium A’ (*iceA*) gene is another virulence factor of relevance [11]. It has two main allelic variants, *iceA1* and *iceA2*. Allele *iceA1* shows sequence homology with a gene from *Neisseria lactamica*, *nlaIIIR*, which encodes a CTAG-specific restriction endonuclease. Furthermore, it has been associated with peptic ulcer disease. On the other hand, *iceA2* has been associated with gastritis, although homology to other genes has not been described and the function of its product remains unclear [12].

Flagella, urease, lipopolysaccharide (LPS) and peptidoglycan are other critical factors related to the pathogenicity of *H. pylori*. Flagella confer motility to the cell and are considered an absolutely essential virulence factor for colonization of the gastric mucosa. Although the presence of the flagellum alone has been shown to be insufficient for colonization, non-mobile mutants lacking the flagellum are unable to establish an infection in animal models [13]. Urease enzyme transforms urea into ammonium and bicarbonate to counteract the acidic environment of the stomach, and it plays an important role in the pathogenesis of *H. pylori*, being correlated with its adhesion and its immunogenicity [13]. Regarding the LPS, its structural characteristics provide the cell with two mechanisms of persistence in its niche. On the one hand, Lewis antigens present in the O-antigen mimic the glycan structures of human cells, thus facilitating the immune escape. On the other hand, the lipid A unique structure provides cell resistance to host cationic antimicrobial peptides (CAMPs) [14,15]. Specifically for *H. pylori*, it has been proposed that the coordinated action of different proteins causes a crosslinking relaxation of the peptidoglycan, which confers its characteristic helical curvature, necessary for reliable bacterial colonization of the stomach [13,15].

Adhesins, bacterial proteins with host cell adhesive properties, are also considered as virulence factors, since they mediate in almost all bacterial pathogenesis [16]. *H. pylori* expresses multiple proteins that act as epithelial cell adhesins, such as BabA, OipA or SabA. One of the most studied adhesins is the protein encoded by *babA2*, BabA, which binds to Lewis b blood-group antigen (Le^b^) found on gastric epithelial cells. Not all *H. pylori* strains express BabA. In those expressing BabA, a correlation with increased risk for gastric cancer has been found [17]. Another *H. pylori* adhesin is OipA, whose expression induces inflammation and cause IL-8 cytokine secretion in host cells. However, the host receptor for OipA has not been yet identified [18]. On the other hand, SabA binds to the sialylated glycan Lex. The capability of many *H. pylori* strains to adhere to sialylated glycoconjugates expressed during chronic inflammation might, therefore, strengthen virulence and the extraordinary chronicity of *H. pylori* infection [19].

Further virulence factors identified in *H. pylori* are outer membrane proteins (OMPs), phospholipids, glycolipids, a γ-glutamyl transpeptidase (GGT) associated with colonization and cell apoptosis, the duodenal ulcer-promoting gene A (*dupA*), and other adhesins (*hpA*, *napA*, etc.) [10,20,21].

The high number of these factors and allelic variation of the involved genes generates a highly complex scenario and reveals the difficulties in testing the contribution of each individual factor. The association of gastric cancer with the *H. pylori* infection has led to its systematic eradication. However, the progression from infection to the development of cancer continues to be unclear, promoting great interest in clarifying this issue. The aim of this study was to investigate possible relevant differences among genomes of strains isolated from tumoral gastric tissue and from non-tumoral tissue. Both samples were obtained from the same patient who suffered from gastric cancer.

## 2. Results

### 2.1. B508A-S1, B508A-T2A, and B508A-T4 Genome Comparison

Basic characteristics of these genomes are shown in Table 1. The average genomic GC content for each strain was 39%, perfectly fitting with the standards of the species, which range from 38% to 39% [22]. The genome length of B508A-S1, B508A-T2A and B508A-T4 was 1,580,553 bp, 1,578,500 bp and 1,585,256 bp, respectively. The total number of genes was 1577 for B508A-S1 and B508A-T2A, and 1581 for B508A-T4. The genomes of B508A-S1, B508A-T2A and B508A-T4 contained a total of 1445, 1442 and 1444 proteins or coding genes, respectively. On average, 624-fold genome coverage of sequence data was used to create draft genome assemblies using the a5 assembler. The number of scaffolds per genome in each assembly ranged from 25 to 45 (average: 35.6 scaffolds) and a median N50 of 143,796 bp. Using Roary, a total of 1456 clusters of orthologous sequences were identified as core genes (>95% of the strains) and 86 were just present in a subset of isolates. The whole gene set, defined as the pangenome, included 1542 genes.

The average nucleotide identity (ANI) values were higher than 99.50% in all comparisons, as expected for strains of the same species. The most similar strains, with an ANI value of 99.94%, were B508A-S1 and B508A-T4. Regarding the B508A-T2A isolate, it had an ANI value of 99.58% with the two other strains.

**Figure 1:**
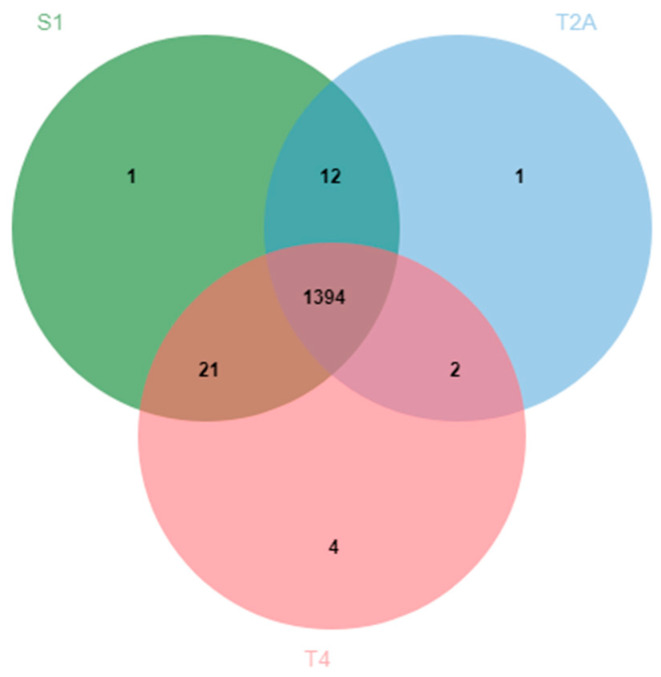
Venn diagram of the three genomes obtained with orthoVenn showing the number of clusters of orthologous genes.

Comparison of the three genomes using OrthoVenn2 showed 1435 clusters of orthologous genes, where 1394 were shared among all strains, two between B508A-T2A and B508A-T4, 21 between B508A-T4 and B508A-S1, and 12 between B508A-S1 and B508A-T2A (Figure 1). The differences among them were mainly in outer membrane proteins, methylases and flagellar biosynthesis proteins (Table S1). Regarding unique clusters, strains B508A-S1 and B508A-T2A had just one, while strain B508A-T4 had four. Each unique cluster had a double copy of the protein. The four unique clusters of B508A-T4 consisted of two Hop family outer membrane proteins, one transposase and one hypothetical protein. The unique cluster of B508A-T2A corresponded to an outer membrane beta-barrel protein. Additionally, the unique cluster of B508A-S1 was found to be a restriction endonuclease. In total, B508A-S1, B508A-T2A and B508A-T4 showed 1428, 1409 and 1421 gene clusters, respectively (Figure 1).

**Figure 2:**
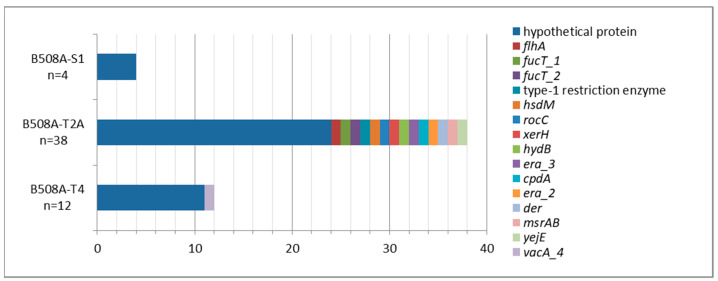
Relative distribution of unique genes found with Roary.

Focusing on the analysis of the unique genes using Roary, the genomes of B508A-T2A, B508A-T4 and B508A-S1 had 38, 12 and four unique genes, respectively, most of them codifying hypothetical proteins with unknown function. A graphical representation of the distribution of unique genes from the comparison of the three genomes is shown in Figure 2. The complete annotation of unique genes with known function can be found in the Table S2.

Nucleotide polymorphisms and indels between genomes were obtained using Snippy. On the one hand, few variants, 169 in total, were found between B508A-S1 and B508A-T4. On the other hand, the number of variants between B508A-T2A and the other genomes was higher, specifically 3344 with B508A-S1 and 3316 with B508A-T4 (Table 2). This fact reflects the special distinction of the strain B508A-T2A with the other strains. Most of the variability was found in coding genes with unknown function (data not shown). Regarding the coding genes with known function, the greatest variability was found in flagellar proteins, proteins of LPS biosynthesis and OMPs.

The circular representation of genomes obtained with the comparative genomic tool CG-View (Circular Genome Viewer) is available in Figure S1. They are markedly similar among them, without visual difference being detected on this representation. It would be necessary to significantly zoom-in the image in order to perceive the minor differences.

The functional distribution of the genomes was obtained with the Rapid Annotation Subsystem Technology (RAST) server (Figure S2). It showed the same subsystem distribution statistics for all the strains, with few differences in the categories of DNA and protein metabolism, motility, and chemotaxis. Remarkably, in genome B508A-T2A, a unique protein was found, which was classified as a type I restriction modification system, specifically subunit S (EC 3.1.21.3), which could be of interest because it is present on the strain with more virulence determinants, as described in next section.

### 2.2. Virulence Factors and Antimicrobial Resistances

The analysis of the results from Diamond shows that all three strains contain all the urease genes, the flagella forming genes and most of the virulence factors analyzed, including *vacA* and *iceA* (Table 3). By contrast, they are *babA/hopS* and *sabB/hopO* negative. There are some differences in the number of copies in the *babB/hopT* adherence gene and in the genes involved in immune evasion *(futA*, *futB* and *futC* genes). All isolates have the *vacA i2/m2* genotype (Table 1). Furthermore, on the one hand, the strain B508A-T2A has the *s1* allele. On the other hand, the isolates B508A-S1 and B508A-T4 are *vacA s2*, as evidenced by the size of the fragment within the primers (Figure S3). It should be noted that conventional polymerase chain reactions (PCRs) could not discriminate between alleles. None of the three isolates has the *cag*PAI nor any other genomic island (Table 1).

Possible CRISPR (clustered regularly interspaced short palindromic repeats) sequences were sought using CRISPR-Finder. No confirmed sequence was found, only two questionable ones in each of the genomes. The sequences of the two spacers were identical in all cases and located in the middle of a coding gene which was annotated as a toxin. The locus tags for B508A-S1, B508A-T2A and B508A-T4 were DDP45_03600, DDP35_03380 and DDP36_03845, respectively. Furthermore, PILER-CR—tool for rapid identification and classification of CRISPR repeats—could not find any putative CRISPR array.

No resistance genes were found using the database ResFinder. The sequences of genes *pbp1* (amoxicillin), 23S rRNA (clariythromicin), 16S rRNA (tetracycline), *frxA, rdxA* (metronidazole) and *gyrA* (levofloxacin) were manually checked to confirm this statement and, indeed, no mutation responsible for the resistance was found (Table 4).

### 2.3. H. pylori Genetic Population

**Figure 3:**
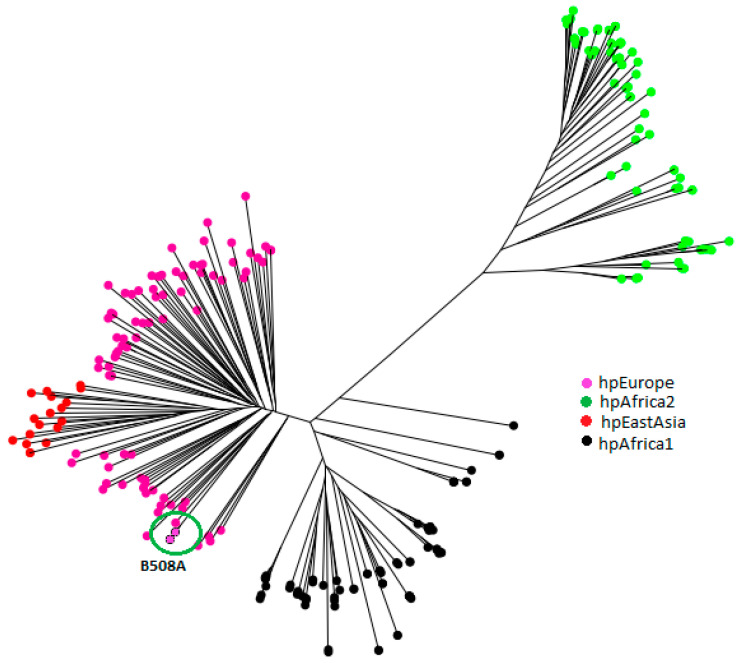
Phylogenetic tree showing the position of the three strains within the hpEurope population.

In the phylogenetic tree obtained with MLST (multilocus sequence typing) analysis, four of the actual populations can be observed. The most distant is hpAfrica2, due to its ancestral evolution. Population hpAfrica1 is clearly separated from hpAfrica2 and the other ones. The population hpEastAsia expands from within the hpEurope population, showing the fact that East Asian *H. pylori* diverged from European lineages, as suggested by Kawai et al. [23]. Finally, circled in this phylogenetic tree are the three strains of this study, which lie within the hpEurope population (Figure 3).

## 3. Discussion

Even though hundreds of *H. pylori* genomes have been published, few studies focused on different strains obtained from the same patient at a single point in time [24]. Recently, we suggested that the predominant pattern of *H. pylori* infections in humans are events of multiple infections, including a predominant strain and multiple minority *H. pylori* strains [25].

This study focused directly on three strains, isolated from a patient suffering gastric cancer. The aforementioned study showed, through amplicon sequencing of housekeeping genes, that these three strains were a result of microevolution events by mutation of an original strain infecting the gastric mucosa. Additionally, the interest on using these strains lies in the finding that the strain B508A-T4 (isolated from tumoral tissue) was found genetically closer to the strain B508A-S1 (from non-tumoral tissue) than to the strain B508A-T2A (from the same tumoral tissue).

The ANI values of the B508 isolates were ≥99.58%, being consistent with the definition of species [26], and it is even more consistent considering they come from microevolution events. As shown by Palau et al. [25,27], the strain B508A-T2A is the most distant among them. Therefore, the aim of the study was to search within these three strains for genetic markers that could be linked with the level of virulence, pathogenicity or the risk of developing gastric cancer.

The strain B508A-T2A is the most divergent isolate, as seen by multiple factors. One of them is the length of its genome, being the smallest of the three with a difference of 2053 bp with B508A-S1 and 6756 bp with B508A-T4, respectively (Table 1). It also has fewer coding genes than the rest, indicating a gene loss. Any change in selection pressure might contribute to a gene loss, which might be done in several ways: selection for a smaller genome, some genes become actively deleterious, or some genes become less necessary [28]. Adoption of a pathogenic lifestyle can lead to recurrent changes in selection pressures, due to the host adaptive immune responses [29,30]. Such changes have been observed to occur even within the same strain during the course of infection [31]. Hence, the present results are consistent with the events of microevolution.

Unique genes obtained with Roary (Figure 2) show the relative abundance of the unique genes found in the comparison among the three isolates. The strain B508A-T2A is the one with more unique genes. It can clearly be seen that the area describing hypothetical proteins is much bigger than the rest, showing there are many undiscovered functions. Strain B508A-T4 showed a unique vacuolating cytotoxin autotransporter, although caution must be taken when assigning its real function, because just a small percentage of these proteins have been proved to be able to translocate proteins [32]. Consequently, further studies have to be undertaken in this direction to understand the role of this protein.

As seen in our results, RAST and OrthoVenn analyses point at restriction modification systems, which have been described to have a relevant role in the regulation of gene expression and in modulating virulence [33]. These restriction modification systems are important providers of defense against foreign DNA. Furthermore, in order to avoid destroying its own DNA, methyl groups are added to the sequences by methyltransferases, which also appear in our results.

Also important to mention is that the number of shared genes of strain B508-T2A with any of the two other strains is lower than the number of shared genes between these two other strains (Figure 1), thus, making strain B508-T2A a more differentiated strain.

After studying all the types of polymorphisms among the three strains, this work continued with the focus on the differences between the most different strain, B508A-T2A, and the other two. Specifically, nonsense polymorphisms were sought. Being B508A-T2A the most differentiated strain and isolated from tumoral tissue, the search for these differences serves to highlight possible signs of differentiations between virulent and none-virulent strains. Even though only one strain is here considered, the current results provide hints to narrow the search for possible candidates.

Changes in the expression of bacterial surface structures, such as OMPs, are anticipated to facilitate adaption of the bacterium to the new human host [34]. A total of 21 non-synonymous or missense single nucleotide polymorphisms (SNPs) were related to outer membrane proteins. Specifically, on the *hof* family of adhesins, six were situated in the *hofB* gene, two in the *hofG* gene and one in the *hofH* gene. The other 12 involved *bamA*, an outer membrane protein assembly factor. Additionally, eight non-synonymous SNPs were detected on the *fliI* gene and a single one in the *flgG* gene. These differences may be of relevance since colonization is the basis of the inflammatory reaction induced by *H. pylori* [35]. Consequently, the motility of *H. pylori*, and in particular the flagellum, is a critical colonization determinant that affects the infection outcome. 

The LPS of this organism plays a key role in its colonization and persistence in the stomach. In addition, the LPS of *H. pylori* modulates pathogen-induced host inflammatory responses. These responses may result in chronic inflammation within the gastrointestinal tract. Very little is known about the LPS compositions of different strains of *H. pylori* with varied degree of virulence in human [36]. Here, 27 missense SNPs in the same LPS biosynthesis protein, a glycosyltransferase, were found in the strain B508A-T2A when comparing it with the two other strains.

Different virulence factors have been described until now, related to persistent colonization of the gastric mucosa, toxin expression or immune evasion [10,37,38]. Some of these pathogenicity factors have been associated with increased risk of gastric cancer: *cagA+* and *vacA s1i1m1* genotypes and the protein expression of AlpA, OipA, BabA, and SabA [39,40,41]. The three studied genomes are *cagA*, *babA* and *sabB/hopO* negative. This is a surprising result for two main reasons. First, as mentioned above, these genes have been clearly defined in the literature as gastric cancer markers and, second, the strains B508A-T2A and B508A-T4 were isolated from tumoral tissue. 

Strain B508A-T2A is the only presenting the genotype *vacA s1*, showing a specific feature directly linked with pathogenicity, while the others are *s2*. On the other hand, the three strains have the coding genes for the proteins AlpA, OipA and SabA and other genes related to pathogenicity (Table 3).

Although CRISPR-Cas systems are of vital importance in the immunological defense of certain bacteria, as they confer resistance to foreign genetic elements, no *cas* genes could be detected in this work, and neither CRISPR-like sequences could be defined. From the bibliography, CRISPR-like loci have been identified in *H. pylori*, but no CRISPR-Cas system has been found so far [42].

Regarding the study of antibiotic resistances, all three strains were defined as susceptible to the five antibiotics studied (amoxicillin, clarithromycin, levofloxacin, metronidazole and tetracycline), as none of them contained any mutation in the sites responsible for the resistances. *H. pylori* is nowadays on the high priority list of the World Health Organization (WHO) for research into and the discovery of new antibiotics. Resistances have arisen in the last years, leading to suboptimal eradication rates [43,44]. Here, however, these three strains show no mutations linked with resistances. This could be explained by multiple factors, such as the patient history of antibiotics intake.

Regarding the MLST analysis and its predicted phylogenetic tree (Figure 3), the three isolates of study were from the group within the hpEurope population, so the European origin of the patient is demonstrated.

## 4. Materials and Methods

### 4.1. H. pylori Strains and Genomic DNA Extraction and Sequencing

The present study focuses on the study of strains B508A-S1, B508A-T2A, and B508A-T4. These were isolated from the stomach of a single patient with adenocarcinoma [27]. In particular, strains B508A-T2A and B508A-T4 were obtained from the same cancerous tissue and strain B508A-S1 from non-tumoral tissue.

The selection and culture of the strains is extensively described in Palau et al. [27]. Briefly, the strains were obtained from the *H. pylori* collection of the Digestive Diseases Department of the Hospital Taulí (Sabadell, Barcelona, Catalonia, Spain). The strains were recovered on Columbia agar with 5% sheep blood (bioMérieux) and subcultured on Columbia blood agar or Brucella agar (BD Diagnostics, Franklin Lakes, NJ, USA) supplemented with 10% fetal bovine serum (Invitrogen, Waltham, MA, USA) at 37 °C under microaerophilic conditions for a week. Different colonies were isolated from antral biopsies B508A-S and B508A-T that were taken from the same patient (B508), B508A-S from normal tissue and B508A-T from gastric adenocarcinoma. Six colonies from each sample were analyzed by multilocus sequence analyses (MLSA) of six housekeeping genes, detecting a unique strain from B508A-S (B508A-S1 = -S2 = -S3 = -S4 = -S5 = -S6), and two different strains from B508A-T, B508A-T2A (= -T2B = -T3 = -T5 = -T6) and B508A-T4.

Genomic DNAs from all three strains were extracted using the genomic DNA extraction kit (REAL, Durviz S.L., València, Spain) following the procedure explained by Palau et al. [27]. Genome sequencing was performed using Illumina technology (San Diego, CA, USA). The sequencing library preparation and sequencing of the whole genome was done by the Centre for Genomic Regulation (CRG, Barcelona, Spain) using an Illumina Hi-Seq Sequencing with 2 × 125 bp v4 chemistry. Following, a de novo assembly of all the set of genomes was carried out using the tool a5-assembler [45]. The alignment and rearrangement of the sequences was achieved using the software Mauve [46]. The draft genome annotation was performed using the NCBI Prokaryotic Genome Annotation Pipeline (PGAP) [47].

### 4.2. Genomic Comparison and Characterization

Pan- and core-genome analyses for all strains were performed using Roary (https://github.com/sanger-pathogens/Roary) (accessed on 4 February 2020) [48].

In order to fully characterize the genomes, different analyses were carried out. Average nucleotide identity (OrthoANIu algorithm) [49] was calculated using EzBioCloud’s ANI calculator (www.ezbiocloud.net/tools/ani) (accessed on 4 February 2020). OrthoVenn2 [50] was used for the comparison of orthologous gene clusters. Snippy [51] was used to find both substitutions and insertions/deletions (indels). Later, snippy-core was used to combine the previous obtained snippy outputs into a core SNP alignment. Snippy and snippy-core were run on the Galaxy platform [52] with the default parameters.

In order to visualize the results, CGView server [53] was used to obtain a circular representation of the genomes. Additionally, the RAST and SEED [54] servers were used to compare the subsystem distribution statistics among all strains.

### 4.3. Virulence and Antimicrobial Resistance Genes

With the aim of finding pathogenic genes, Diamond [55] was used as a search tool against the Virulence-Factor database (VFDB). In addition, PCR was used to determine *H. pylori cagA* status and to genotype the *vacA* gene (signal, intermediate and mid-region polymorphisms), as described by Lario et al. [56]. PCR results were verified by looking for the primers in the sequence of the whole genomes and calculating the size of the fragments to determine the corresponding allele [7,9,57] (Figure S3). Furthermore, PILER-CR [58] and CRISPRCasFinder [59] were used for CRISPR identification.

ResFinder [60] was used to identify acquired antimicrobial resistance genes in the total sequenced isolates of bacteria. Sequences of genes involved in amoxicillin, clarithromycin, tetracycline, metronidazole and levofloxacin resistances were also manually checked: *pbp1* [61,62,63], 23S rRNA [64,65,66,67], 16S rRNA [68,69], *frxA* [70], *rdxA* [71,72] and *gyrA* [73,74], respectively.

### 4.4. H. pylori Genetic Population Assignment

For multilocus sequence typing (MLST) of the three genomes, seven housekeeping genes (*atpA*, *efp*, *mutY*, *ppa*, *trpC*, *ureI* and *yphC*) were extracted using BLAST and the JGI-database (https://img.jgi.doe.gov/) (accessed on 23 April 2019). A total of 243 representative sequences from different *H. pylori* populations were downloaded with public databases PubMLST (https://pubmlst.org/helicobacter/) (accessed on 10 April 2019), and the multiple sequence alignment program MAFFT (https://mafft.cbrc.jp/alignment/server/) (accessed on 23 April 2019) was used to align all the sequences and to predict a neighbor-joining phylogenetic tree.

## 5. Conclusions

With all the accumulated information no significant differences were found between the isolates regarding the virulence and the origin of the isolates. Still, considering that B508A-S1 and B508A-T4 are more closely related and that they originate from non-tumoral and tumoral tissue, respectively, it could be inferred that their characteristics/genes are not a cause for developing a cancerous process. On the other hand, regarding B508A-T2, being markedly distant from the other two and having been found in the tumoral tissue, it can be hypothesised that some of its characteristics could be linked to the pathogenicity of *Helicobacter pylori*. A further consideration to take into account is that even though two strains were extracted from a cancerous tissue, they are all missing some relevant virulence genes such as *cagA*, *babA* or *sabB.* Nonetheless, more exhaustive analyses are needed.

## Figures and Tables

**Table 1:** Main features of the analysed *H. pylori* genomes.

Genome Characteristics	B508A-S1	B508A-T2A	B508A-T4
GC%	39.0	39.0	38.9
Genome Size (bp)	1,586,749	1,584,784	1,585,256
N50	200,200	106,266	124,923
Genes (total)	1577	1577	1581
Genes (unique)	2	13	3
CDS (total)	1535	1535	1536
CDS (coding)	1445	1442	1444
rRNAs (5S, 16S, 23S)	1, 1, 1	1, 1, 1	2, 1 ^1^, 1
tRNAs	36	36	36
ncRNAs	3	3	3
Pseudo Genes	90	93	92
*vacA* (PCR)	*s1-s2*/*m1-m2*	*s1-s2*/*m1-m2*	*s1-s2*/*m1-m2*
*vacA* (genome)	*s2*/*m2*/*i2*	*s1*/*m2*/*i2*	*s2*/*m2*/*i2*
*cagA*	-	-	-
GenBank Accession	QDJN00000000	QDJM00000000	QDJL00000000

^1^ There is one complete sequence (1500 bp) and two partial sequences (circa. 150 bp).

**Table 2:** Nucleotide polymorphisms between genomes.

Type	Number of Polymorphisms		
	B508A-S1 Versus -T4	B508A-S1 Versus -T2	B508A-T2 Versus -T4
SNP ^1^	113	2454	2418
MNP ^2^	10	198	201
Complex	29	618	619
Deletions	12	35	38
Insertions	5	39	40
Total	169	3344	3316

^1^ Single nucleotide polymorphism, ^2^ Multi-nucleotide polymorphism.

**Table 3:** Virulence factors analyzed with Diamond. Gene copy number is indicated.

Virulence Factors	Related Genes	B508A-S1	B508A-T2A	B508A-T4
Urease	^1^	1	1	1
Flagella	^2^	1	1	1
Lipopolysaccharide Lewis antigens	*futA*	1	2	1
	*futB*	1	-	1
	*futC*	2	2	1
Neutrophil-activating protein	*napA*	1	1	1
	*oipA/hopH*	1	1	1
Vacuolating cytotoxin	*vacA*	1	1	1
Adherence-associated lipoproteins	*alpA/hopC*	1	1	1
	*alpB/hopB*	1	1	1
Blood group antigen binding adhesins	*babA/hopS*	-	-	-
	*babB/hopT*	3	2	3
*H. pylori* adhesin A	*hpaA*	1	1	1
HopZ	*hopZ*	1	1	1
HorB	*horB*	1	1	1
Sialic acid binding proteins	*sabA/hopP*	1	1	1
	*sabB/hopO*	-	-	-
Duodenal ulcer promoting	*dupA*	1	1	1
Induced by contact with epithelium A ^3^	*iceA*	1	1	1

^1^ Include all these genes: *ureA, ureB, ureI, ureE, ureF, ureG, ureH*, ^2^ Include all these genes: *flaA, flaB, flaG, fliR, flgI, flgL, flgH, flaG, fliF, fliG, fliH, flgG_1, flhA, flhF, fliA, fliM, fliY, fliN, fliP, fliD, fliS, flhB_1, fliL, motA, motB, flgE_1, flgD, flgE_2, flgK, flA, fliQ, fliI, flgA, fliE, flgC, flgB, flhB_2, flgG_2*, ^3^ Found with JGI IMG (https://img.jgi.doe.gov/ accessed on 10 April 2019).

**Table 4:** Nucleotides/amino acids found in the described mutational positions in resistance genes for each antibiotic.

Gene	Mutational Position	Susceptible Strains	B508A-S1	B508A-T2	B508A-T4
*pbp1*	V374L	V	V	V	V
	E406A	E	E	E	E
	S414R	S	S	S	S
	T593A	T	T	T	T
	A599G	A	A	A	A
	V601G	V	V	V	V
23S rRNA	A2143G	A	A	A	A
	A2144G	A	A	A	A
	T2183C	T	T	T	T
	C2196T	C	C	C	C
	A2224G	A	A	A	A
16S rRNA	A926T	A	A	A	A
	G927T	G	G	G	G
	A928C	A	A	A	A
*frxA*	A32V	A	A	A	A
	A70T	A	A	A	A
	F72S	F	F	F	F
	G73S	G	G	G	G
	A138V	A	A	A	A
	A152V	A	A	A	A
	A153V	A	A	A	A
	C193S	C	C	C	C
*rdxA*	G3A	G	G	G	G
	C46T	C	C	C	C
	G238A	G	G	G	G
	G352A	G	G	G	G
*gyrA*	D86N	D	D	D	D
	N87K\I\Y	N	N	N	N
	A88V	A	A	A	A
	D91Y\G\N	D	D	D	D

## Data Availability

MDPI Research Data Policies at https://www.mdpi.com/ethics (accessed on 11 March 2021).

## Supplementary Materials

The following are available online at https://www.mdpi.com/2076-0817/10/3/331/s1, Figure S1: circular comparative representation of the whole genomes using CGView, Figure S2: pie charts representing Rapid Annotation Subsystem Technology (RAST) functional subsystems in the B508A-S1 (a), B508A-T2A (b), and B508A-T4 (c) genomes, Figure S3: vacA genomic sequences extracted from the whole genomes of strains B508A-S1, B508A-T2A and B508A-T4, Table S1: shared clusters between strains found using OrthoVenn2, Table S2: Description of the unique genes found with Roary.

## References

[1] Baltrus D.A., Blaser M.J., Guillemin K. (2009). *Helicobacter pylori* genome plasticity. Genome Dyn..

[2] Chiurillo M.A., Moran Y., Cañas M., Valderrama E., Granda N., Sayegh M., Ramírez J.L. (2013). Genotyping of *Helicobacter pylori* virulence-associated genes shows high diversity of strains infecting patients in Western Venezuela. Int. J. Infect. Dis..

[3] Gressmann H., Linz B., Ghai R., Pleissner K.-P., Schlapbach R., Yamaoka Y., Kraft C., Suerbaum S., Meyer T.F., Achtman M. (2005). Gain and loss of multiple genes during the evolution of *Helicobacter pylori*. PLoS Genet..

[4] You Y., He L., Zhang M., Zhang J. (2015). Comparative genomics of a *Helicobacter pylori* isolate from a Chinese Yunnan Naxi ethnic aborigine suggests high genetic divergence and phage insertion. PLoS ONE.

[5] Cao D.M., Lu Q.F., Li S.B., Wang J.P., Chen Y.L., Huang Y.Q., Bi H.K. (2016). Comparative genomics of *H. pylori* and non-pylori *Helicobacter* species to identify new regions associated with its pathogenicity and adaptability. Biomed. Res. Int..

[6] Junaid M., Linn A.K., Javadi M.B., Al-Gubare S., Ali N., Katzenmeier G. (2016). Vacuolating cytotoxin A (VacA)—A multi-talented pore-forming toxin from *Helicobacter pylori*. Toxicon.

[7] Atherton J.C., Cao P., Peek R.M., Tummuru M.K.R., Blaser M.J., Cover T.L. (1995). Mosaicism in vacuolating cytotoxin alleles of *Helicobacter pylori*. Association of specific *vacA* types with cytotoxin production and peptic ulceration. J. Biol. Chem..

[8] Rudi J., Kolb C., Maiwald M., Kuck D., Sieg A., Galle P.R., Stremmel W. (1998). Diversity of *Helicobacter pylori vacA* and *cagA* genes and relationship to VacA and CagA protein expression, cytotoxin production, and associated disease. J. Clin. Microbiol..

[9] Rhead J.L., Letley D.P., Mohammadi M., Hussein N., Mohagheghi M.A., Eshagh Hosseini M., Atherton J.C. (2007). A new *Helicobacter pylori* vacuolating cytotoxin determinant, the intermediate region, is associated with gastric cancer. Gastroenterology.

[10] Mascellino M.T., Margani M., Oliva A. (2009). *Helicobacter pylori*: Determinant and markers of virulence. Dis. Markers.

[11] Peek R.M., Thompson S.A., Atherton J.C., Blaser M.J., Miller G.G. (1996). Expression of a novel ulcer-associated *H. pylori* gene, *iceA*, following adherence to gastric epithelial cells. Gastroenterology.

[12] da Costa D.M., dos Santos Pereira E., Rabenhorst S.H.B. (2015). What exists beyond *cagA* and *vacA*? Helicobacter pylori genes in gastric diseases. World J. Gastroenterol..

[13] Piqué N., Palau M., Berlanga M., Miñana-Galbis D. (2016). Advances in the research of new genetic markers for the detection of *Helicobacter pylori* infection. Recent Advances in Pharmaceutical Sciences VI.

[14] Sycuro L.K., Pincus Z., Gutierrez K.D., Biboy J., Stern C.A., Vollmer W., Salama N.R. (2010). Peptidoglycan crosslinking relaxation promotes *Helicobacter pylori*’s helical shape and stomach colonization. Cell.

[15] Li H., Liao T., Debowski A.W., Tang H., Nilsson H.O., Stubbs K.A., Marshall B.J., Benghezal M. (2016). Lipopolysaccharide structure and biosynthesis in *Helicobacter pylori*. Helicobacter.

[16] Patel S., Mathivanan N., Goyal A. (2017). Bacterial adhesins, the pathogenic weapons to trick host defense arsenal. Biomed. Pharmacother..

[17] Israel D.A., Peek R.M. (2010). Surreptitious manipulation of the human host by *Helicobacter pylori*. Gut Microbes.

[18] Teymournejad O., Mobarez A.M., Hassan Z.M., Talebi Bezmin Abadi A. (2017). Binding of the *Helicobacter pylori* OipA causes apoptosis of host cells via modulation of Bax/Bcl-2 levels. Sci. Rep..

[19] Mahdavi J., Sondén B., Hurtig M., Olfad F.O., Forsberg L., Roche N., Angstrom J., Larsson T., Teneberg S., Karlsson K.A. (2002). *Helicobacter pylori* SabA adhesin in persistent infection and chronic inflammation. Science.

[20] Mobley H.L.T., Mendz G.L., Hazell S.L. (2001). Helicobacter pylori: Physiology and Genetics.

[21] Backert S., Clyne M. (2011). Pathogenesis of *Helicobacter pylori* infection. Helicobacter.

[22] Ali A., Naz A., Soares S.C., Bakhtiar M., Tiwari S., Hassan S.S., Hanan F., Ramos R., Pereira U., Barh D. (2015). Pan-genome analysis of human gastric pathogen *H. pylori*: Comparative genomics and pathogenomics approaches to identify regions associated with pathogenicity and prediction of potential core therapeutic targets. Biomed. Res. Int..

[23] Kawai M., Furuta Y., Yahara K., Tsuru T., Oshima K., Handa N., Takahashi N., Yoshida M., Azuma T., Hattori M. (2011). Evolution in an oncogenic bacterial species with extreme genome plasticity: *Helicobacter pylori* East Asian genomes. BMC Microbiol..

[24] Cao Q., Didelot X., Wu Z., Li Z., He L., Li Y., Ni M., You Y., Lin X., Li Z. (2015). Progressive genomic convergence of two *Helicobacter pylori* strains during mixed infection of a patient with chronic gastritis. Gut.

[25] Palau M., Piqué N., Comeau A.M., Douglas G.M., Ramírez-Lázaro M.J., Lario S., Calvet X., Langille M.G.I., Miñana-Galbis D. (2020). Detection of *Helicobacter pylori* microevolution and multiple infection from gastric biopsies by housekeeping gene amplicon sequencing. Pathogens.

[26] Goris J., Konstantinidis K.T., Klappenbach J.A., Coenye T., Vandamme P., Tiedje J.M. (2007). DNA-DNA hybridization values and their relationship to whole-genome sequence similarities. Int. J. Syst. Evol. Microbiol..

[27] Palau M., Kulmann M., Ramírez-Lázaro M.J., Lario S., Quílez M.E., Campo R., Piqué N., Calvet X., Miñana-Galbis D. (2016). Usefulness of housekeeping genes for the diagnosis of *Helicobacter pylori* infection, strain discrimination and detection of multiple infection. Helicobacter.

[28] Weinert L.A., Welch J.J. (2017). Why might bacterial pathogens have small genomes?. Trends Ecol. Evol..

[29] Brodsky I.E., Medzhitov R. (2009). Targeting of immune signalling networks by bacterial pathogens. Nat. Cell. Biol..

[30] Schulte R.D., Makus C., Schulenburg H. (2013). Host-parasite coevolution favours parasite genetic diversity and horizontal gene transfer. J. Evol. Biol..

[31] Draper J.L., Hansen L.M., Bernick D.L., Abedrabbo S., Underwood J.G., Kong N., Huang B.C., Weis A.M., Weimer B.C., van Vliet A.H.M. (2017). Fallacy of the unique genome: Sequence diversity within single *Helicobacter pylori* strains. MBio.

[32] Fischer W., Buhrdorf R., Gerland E., Haas R. (2001). Outer membrane targeting of passenger proteins by the vacuolating cytotoxin autotransporter of *Helicobacter pylori*. Infect. Immun..

[33] Ershova A.S., Rusinov I.S., Spirin S.A., Karyagina A.S., Alexeevski A.V. (2015). Role of restriction-modification systems in prokaryotic evolution and ecology. Biochemistry (Mosc.).

[34] Linz B., Windsor H.M., Gajewski J.P., Hake C.M., Drautz D.I., Schuster S.C., Marshall B.J. (2013). *Helicobacter pylori* genomic microevolution during naturally occurring transmission between adults. PLoS ONE.

[35] Gu H. (2017). Role of flagella in the pathogenesis of *Helicobacter pylori*. Curr. Microbiol..

[36] Leker K., Lozano-Pope I., Bandyopadhyay K., Choudhury B.P., Obonyo M. (2017). Comparison of lipopolysaccharides composition of two different strains of *Helicobacter pylori*. BMC Microbiol..

[37] Kim I.-J., Blanke S.R. (2012). Remodeling the host environment: Modulation of the gastric epithelium by the *Helicobacter pylori* vacuolating toxin (VacA). Front Cell. Infect. Microbiol..

[38] Salama N.R., Hartung M.L., Müller A. (2013). Life in the human stomach: Persistence strategies of the bacterial pathogen *Helicobacter pylori*. Nat. Rev. Microbiol..

[39] Su Y.-L., Huang H.-L., Huang B.-S., Chen P.-C., Chen C.-S., Wang H.-L. (2016). Combination of OipA, BabA, and SabA as candidate biomarkers for predicting *Helicobacter pylori*-related gastric cancer. Sci. Rep..

[40] Berthenet E., Yahara K., Thorell K., Pascoe B., Meric G., Mikhail J.M., Engstrand L., Enroth H., Burette A., Megraud F. (2018). A GWAS on *Helicobacter pylori* strains points to genetic variants associated with gastric cancer risk. BMC Biol..

[41] Pormohammad A., Ghotaslo R., Leylabadlo H.E., Nasiri M.J., Dabiri H., Hashemi A. (2018). Risk of gastric cancer in association with *Helicobacter pylori* different virulence factors: A systematic review and meta-analysis. Microb. Pathog..

[42] García-Zea J.A., de la Herrán R., Rodríguez F.R., Navajas-Pérez R., Rejón C.R. (2019). Detection and variability analyses of CRISPR-like loci in the *H. pylori* genome. PeerJ..

[43] Alba C., Blanco A., Alarcón T. (2017). Antibiotic resistance in *Helicobacter pylori*. Curr. Opin. Infect. Dis..

[44] World Health Organization (WHO) Global Priority List of Antibiotic-Resistant Bacteria to Guide Research, Discovery, and Development of New Antibiotics. https://www.who.int/medicines/publications/WHO-PPL-Short_Summary_25Feb-ET_NM_WHO.pdf?ua=1.

[45] Coil D., Jospin G., Darling A.E. (2015). A5-miseq: An updated pipeline to assemble microbial genomes from Illumina MiSeq data. Bioinformatics.

[46] Darling A.C.E., Mau B., Blattner F.R., Perna N.T. (2004). Mauve: Multiple alignment of conserved genomic sequence with rearrangements. Genome Res..

[47] Tatusova T., Dicuccio M., Badretdin A., Chetvernin V., Nawrocki E.P., Zaslavsky L., Lomsadze A., Pruitt K.D., Borodovsky M., Ostell J. (2016). NCBI prokaryotic genome annotation pipeline. Nucleic Acids Res..

[48] Page A.J., Cummins C.A., Hunt M., Wong V.K., Reuter S., Holden M.T.G., Fookes M., Falush D., Keane J.A., Parkhill J. (2015). Roary: Rapid large-scale prokaryote pan genome analysis. Bioinformatics.

[49] Lee I., Kim Y.O., Park S.C., Chun J. (2016). OrthoANI: An improved algorithm and software for calculating average nucleotide identity. Int. J. Syst. Evol. Microbiol..

[50] Xu L., Dong Z., Fang L., Luo Y., Wei Z., Guo H. (2019). OrthoVenn2: A web server for whole-genome comparison and annotation of orthologous clusters across multiple species. Nucleic Acids Res..

[51] Seemann T. Snippy: Fast Bacterial Variant Calling from NGS Reads. https://github.com/tseemann/snippy.

[52] Afgan E., Baker D., van den Beek M., Blankenberg D., Bouvier D., Čech M., Chilton J., Clements D., Coraor N., Eberhard C. (2016). The Galaxy platform for accessible, reproducible and collaborative biomedical analyses: 2016 update. Nucleic Acids Res..

[53] Stothard P., Grant J.R., Van Domselaar G. (2017). Visualizing and comparing circular genomes using the CGView family of tools. Brief Bioinform..

[54] Overbeek R., Olson R., Pusch G.D., Olsen G.J., Davis J.J., Disz T., Edwards R.A., Gerdes S., Parrello B., Shukla M. (2014). The SEED and the rapid annotation of microbial genomes using subsystems technology (RAST). Nucleic Acids Res..

[55] Buchfink B., Xie C., Huson D.H. (2015). Fast and sensitive protein alignment using DIAMOND. Nat. Methods.

[56] Lario S., Ramírez-Lázaro M.J., Aransay A.M., Lozano J.J., Montserrat A., Casalots Á., Junquera F., Álvarez J., Segura F., Campo R. (2012). MicroRNA profiling in duodenal ulcer disease caused by *Helicobacter pylori* infection in a Western population. Clin. Microbiol. Infect..

[57] Atherton J.C., Cover T.L., Twells R.J., Morales M.R., Hawkey C.J., Blaser M.J. (1999). Simple and accurate PCR-based system for typing vacuolating cytotoxin alleles of *Helicobacter pylori*. J. Clin. Microbiol..

[58] Edgar R.C. (2007). PILER-CR: Fast and accurate identification of CRISPR repeats. BMC Bioinform..

[59] Couvin D., Bernheim A., Toffano-Nioche C., Touchon M., Michalik J., Néron B., Rocha E.P.C., Vergnaud G., Gautheret D., Pourcel C. (2018). CRISPRCasFinder, an update of CRISRFinder, includes a portable version, enhanced performance and integrates search for Cas proteins. Nucleic Acids Res..

[60] Zankari E., Hasman H., Cosentino S., Vestergaard M., Rasmussen S., Lund O., Aarestrup F.M., Larsen M.V. (2012). Identification of acquired antimicrobial resistance genes. J. Antimicrob. Chemother..

[61] Gerrits M.M., Godoy A.P., Kuipers E.J., Ribeiro M.L., Stoof J., Mendonça S., van Vliet A.H., Pedrazzoli J. Jr., Kusters J.G. (2006). Multiple mutations in or adjacent to the conserved penicillin-binding protein motifs of the penicillin-binding protein 1A confer amoxicillin resistance to *Helicobacter pylori*. Helicobacter.

[62] Rimbara E., Noguchi N., Kawai T., Sasatsu M. (2008). Mutations in penicillin-binding proteins 1, 2 and 3 are responsible for amoxicillin resistance in *Helicobacter pylori*. J. Antimicrob. Chemother..

[63] Nishizawa T., Suzuki H., Tsugawa H., Muraoka H., Matsuzaki J., Hirata K., Ikeda F., Takahashi M., Hibi T. (2011). Enhancement of amoxicillin resistance after unsuccessful *Helicobacter pylori* eradication. Antimicrob. Agents Chemother..

[64] Stone G.G., Shortridge D.E.E., Versalovic J., Beyer J., Flamm R.K., Graham D.Y., Ghoneim A.T., Tanaka S.K. (1997). A PCR-Oligonucleotide ligation assay to determine the prevalence of 23S rRNA gene mutations in clarithromycin-resistant *Helicobacter pylori*. Antimicrob. Agents Chemother..

[65] Furuta T., Soya Y., Sugimoto M., Shirai N., Nakamura A., Kodaira C., Nishino M., Okuda M., Okimoto T., Murakami K. (2007). Modified allele-specific primer–polymerase chain reaction method for analysis of susceptibility of *Helicobacter pylori* strains to clarithromycin. J. Gastroenterol. Hepatol..

[66] Agudo S., Pérez-Pérez G., Alarcón T., López-Brea M. (2011). Rapid detection of clarithromycin resistant *Helicobacter pylori* strains in Spanish patients by polymerase chain reaction-restriction fragment length polymorphism. Rev. Española Quimioter..

[67] Mahachai V., Sirimontaporn N., Tumwasorn S., Thong-Ngam D., Vilaichone R.-K. (2011). Sequential therapy in clarithromycin-sensitive and -resistant *Helicobacter pylori* based on polymerase chain reaction. J. Gastroenterol. Hepatol..

[68] Glocker E., Berning M., Gerrits M.M., Kusters J.G., Kist M. (2005). Real-Time PCR screening for 16S rRNA mutations associated with resistance to tetracycline in *Helicobacter pylori*. Antimicrob. Agents Chemother..

[69] Lawson A.J., Elviss N.C., Owen R.J. (2005). Real-time PCR detection and frequency of 16S rDNA mutations associated with resistance and reduced susceptibility to tetracycline in *Helicobacter pylori* from England and Wales. J. Antimicrob. Chemother..

[70] Domanovich-Asor T., Motro Y., Khalfin B., Craddock H.A., Peretz A., Moran-Gilad J. (2020). Genomic analysis of antimicrobial resistance genotype-to-phenotype agreement in *Helicobacter pylori*. Microorganisms.

[71] Kwon D., El-Zaatari F.A.K., Kato M., Osato M.S., Reddy R., Yamaoka Y., Graham D.Y. (2000). Analysis of *rdxA* and involvement of additional genes encoding NAD(P)H flavin oxidoreductase (FrxA) and ferredoxin-like protein (FdxB) in metronidazole resistance of *Helicobacter pylori*. Antimicrob. Agents Chemother..

[72] Binh T.T., Suzuki R., Huyen T., Kwon H., Yamaoka Y. (2015). Search for novel candidate mutations for metronidazole resistance in *Helicobacter pylori* using next-generation sequencing. Antimicrob. Agents Chemother..

[73] Trespalacios-Rangél A.A., Otero W., Arévalo-Galvis A., Poutou-Piñales R.A., Rimbara E., Graham D.Y. (2016). Surveillance of levofloxacin resistance in *Helicobacter pylori* isolates in Bogotá-Colombia (2009–2014). PLoS ONE.

[74] Zerbetto De Palma G., Mendiondo N., Wonaga A., Viola L., Ibarra D., Campitelli E., Salim N., Corti R., Goldman C., Catalano M. (2017). Occurrence of mutations in the antimicrobial target genes related to levofloxacin, clarithromycin, and amoxicillin resistance in *Helicobacter pylori*. Microb. Drug Resist..

