# Supplementary material for: Whole-Genome Sequencing and Comparative Genomics of Three Helicobacter pylori Strains Isolated from the Stomach of a Patient with Adenocarcinoma
Source: Pathogens. 2021 Mar 12;10(3):331. doi: 10.3390/pathogens10030331 (PMC7998635; doi:10.3390/pathogens10030331)
Supplement: Supplementary file 1 [file pathogens-10-00331-s001.pdf]

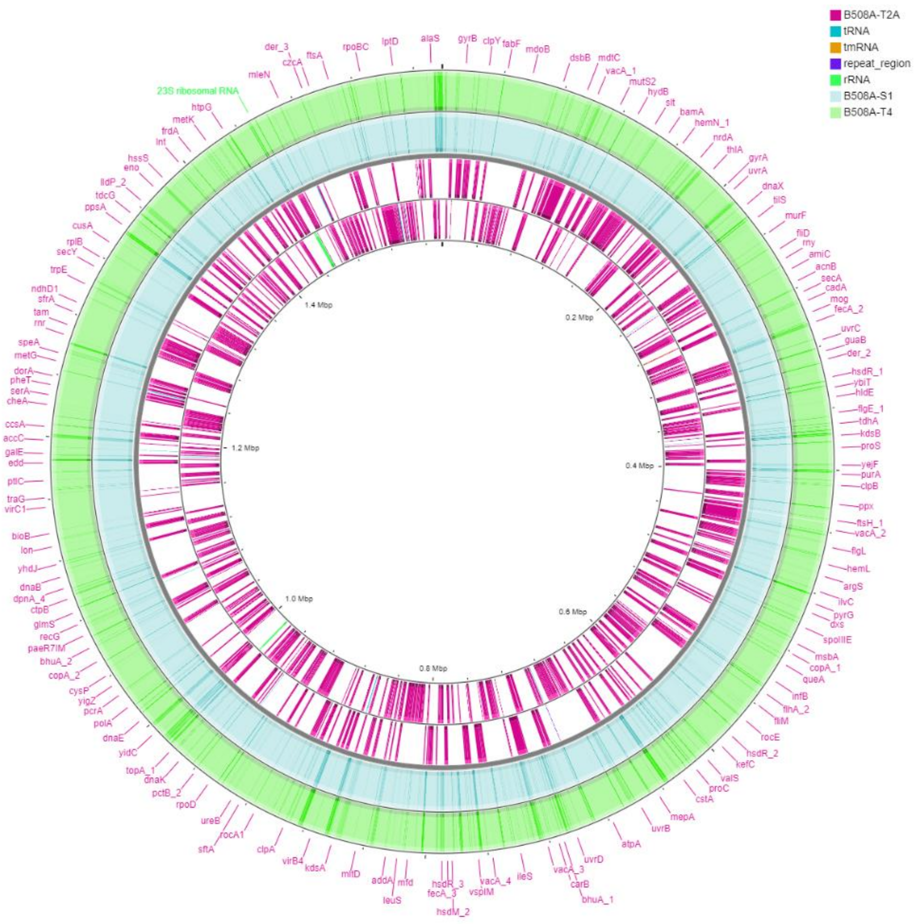

Figure S1. Circular comparative representation of the whole genomes using CGView. Inner circles represent the forward and reverse sequences of B508A-T2A.

(a)

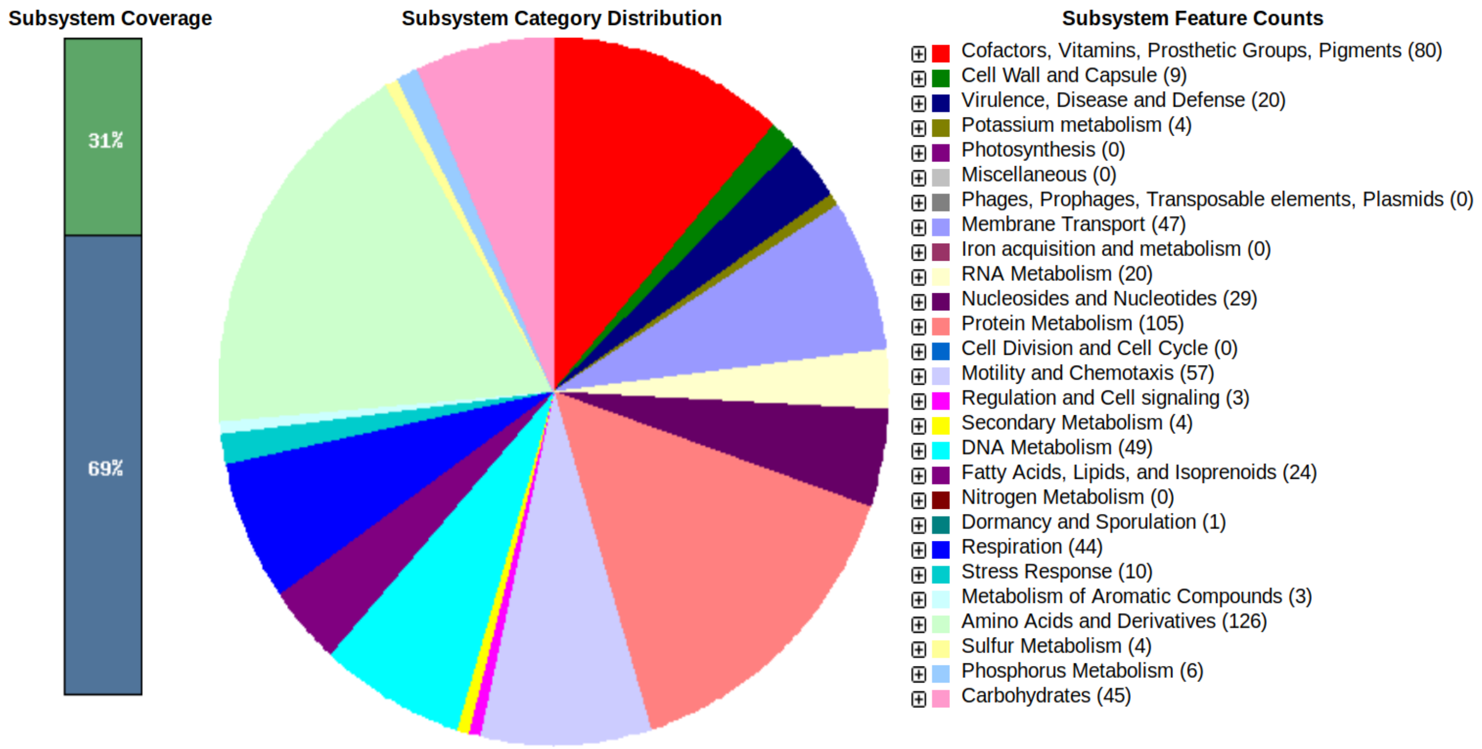
Subsystem Coverage
31%
69%
Subsystem Category Distribution
Subsystem Feature Counts
Cofactors, Vitamins, Prosthetic Groups, Pigments (80)
Cell Wall and Capsule (9)
Virulence, Disease and Defense (20)
Potassium metabolism (4)
Photosynthesis (0)
Miscellaneous (0)
Phages, Prophages, Transposable elements, Plasmids (0)
Membrane Transport (47)
Iron acquisition and metabolism (0)
RNA Metabolism (20)
Nucleosides and Nucleotides (29)
Protein Metabolism (105)
Cell Division and Cell Cycle (0)
Motility and Chemotaxis (57)
Regulation and Cell signaling (3)
Secondary Metabolism (4)
DNA Metabolism (49)
Fatty Acids, Lipids, and Isoprenoids (24)
Nitrogen Metabolism (0)
Dormancy and Sporulation (1)
Respiration (44)
Stress Response (10)
Metabolism of Aromatic Compounds (3)
Amino Acids and Derivatives (126)
Sulfur Metabolism (4)
Phosphorus Metabolism (6)
Carbohydrates (45)

(b)

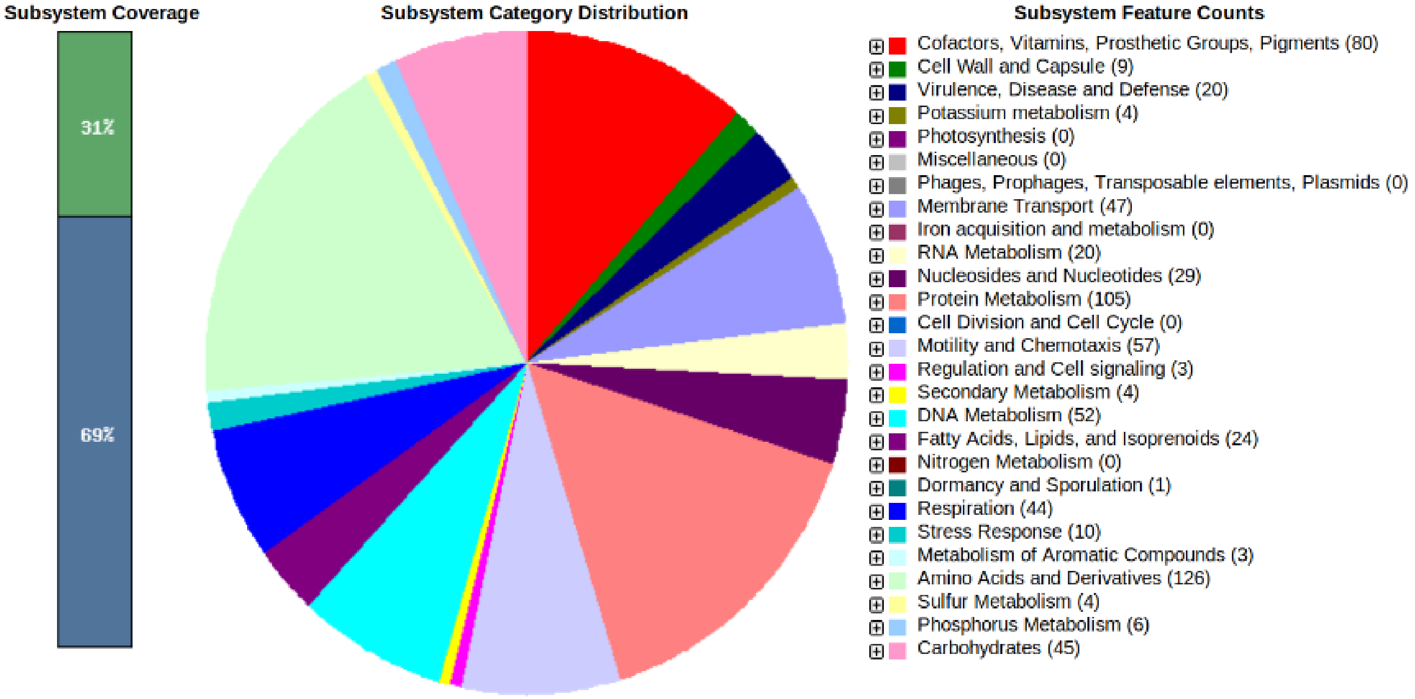

Subsystem Coverage
31%
69%
Subsystem Category Distribution
Subsystem Feature Counts
Cofactors, Vitamins, Prosthetic Groups, Pigments (80)
Cell Wall and Capsule (9)
Virulence, Disease and Defense (20)
Potassium metabolism (4)
Photosynthesis (0)
Miscellaneous (0)
Phages, Prophages, Transposable elements, Plasmids (0)
Membrane Transport (47)
Iron acquisition and metabolism (0)
RNA Metabolism (20)
Nucleosides and Nucleotides (29)
Protein Metabolism (105)
Cell Division and Cell Cycle (0)
Motility and Chemotaxis (57)
Regulation and Cell signaling (3)
Secondary Metabolism (4)
DNA Metabolism (52)
Fatty Acids, Lipids, and Isoprenoids (24)
Nitrogen Metabolism (0)
Dormancy and Sporulation (1)
Respiration (44)
Stress Response (10)
Metabolism of Aromatic Compounds (3)
Amino Acids and Derivatives (126)
Sulfur Metabolism (4)
Phosphorus Metabolism (6)
Carbohydrates (45)

(c)

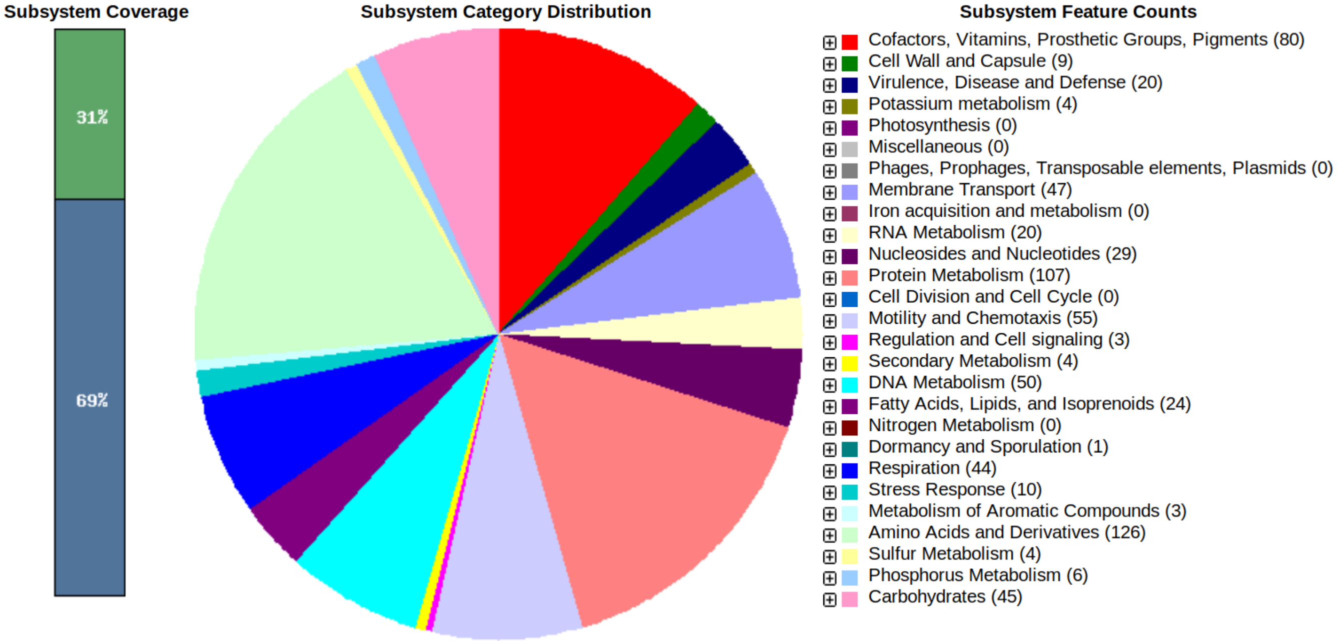

Figure S2. Pie charts representing RAST functional subsystems in the B508A-S1 (**a**), B508A-T2A (**b**), and B508A-T4 (**c**) genomes. The pie chart shows the % distribution of 19 most abundant subsystems on the "category" level, each of which is represented by a particular colour indicated in the right column showing the counts of features.

(a)

B508A-S1 s2m2i2 scaffold_15 (s= 286bp, m= 642bp, i= 432bp)

1 TGCTAAATTT AGCGTTTTGT GCGCTTAATT CTAAATTTTT ACTAAAATCG CTTATTGAGT
61 GGCTTCGCTC TAACGCTCTT TTTAGGTGAT ACTTTAAAGC CGCGCCTGCG GTGGCTTCGT
121 TTAAAGCGTA GCCGCTAAAC TAAAACTTTT TTTAAGAGCG CAGTTGTTGT AAGGCTTCTT
...
5641 ACTTTTTAAT CCTATGGGTT TTATATTCAT TTATCTTAAC TTAATAAAAA TTGAACATTG
5701 GTTGTAGATA CTGCATATTT ATAACCTTAA TCGTAAATGC AACAGAAATT TTCTAGTCTA
5761 AAGTCGCACC CTTTGTGCAA AAATCGTTTT ACAAAAAGAA AGGAAAAAAA TGGAAATACA
5821 ACAAACACAC CGCAAAATCA ATCGCCCCAT TATCTCTCTC GCTCTAGTGG GGGTGTTAAT
5881 GGGCACCGAA CTAGGGGCTA ATACGCCAAA TGATCCCATA CACAGCGAGA GTCGCGCCTT
5941 TTTTACAACC GTGATCATTC CAGCCATTGT TGGGGGTATC GCTACAGGCG CTGCTGTAGG
6001 AACGGTCTCA GGGCTTCTTA GCTGGGGGCT CAAACAAGCC GAACAAGCCA ATAAAGCCCC
6061 AGACAAACCC GATAAAGTTT GGCGCATTCA AGCAGGAAGA GGCTTTGATA ATTTCCCCCA
6121 CAAGCAATAC GACTTATACA AATCCCTACT ATCTAGCAAA ATTGATGGAG GTTGGGACTG
6181 GGGGAATGCC GCTAGGCATT ATTGGGTCAA AGACGGGCAG TGGAACAAAC TTGAAGTGGA
6241 TATGCAAAAC GCTGTAGGGA CTTATAACCT TTCAGGCCTT ATCAACTTTA CTGGTGGGGA
6301 TTTGGACATC AATATGCAAA AAGCCACTTT GCGTTTGGGC CAATTCAATG GCAATTCTTT
6361 CACAAGCTAT AAGGATAGCG CCGATCGCAC CACGAGGGTG AATTTTGACG CTAAAAATAT
6421 CTTAATTGAT AATTTTGTAG AAATCAACAA TCGTGTGGGT TCTGGAGCCG GAAGAAAAGC
6481 CAGCTCTACG GTTTTGACCT TGAAAAGTTC AGAAAAAATT ACAAGCCGTG AAAACGCGGA
6541 AATCTCTCTT TATGATGGCG CCACGCTCAA TTTGGTTTCA AGCTCAAATC AGAGCGTTGA
6601 TCTATATGGG AAAGTGTGGA TGGGCCGTTT GCAATACGTG GGAGCGTATT TAGCCCCTTC
6661 ATACAGCACA ATAGACACTT CAAAAGTGCA AGGGGAAATG AATTTTCGCC ATCTCGCTGT
6721 GGGTGATCAA AACGCCGCTC AAGCGGGCAT TATCGCTAAT AAAAAGACTA ATATTGGCAC
6781 ACTGGATTTG TGGCAAAGTG CGGGGTTAAG CATCATCACC CCTCCCGAAG GCGGTTATGA
6841 GAGTAAAACT AAAGATAACC CTTCTCAAAA CAACCCTAAA AATGACACGC AAAAAACAGA
6901 AATTCAACCC ACGCAAGTCA TTGACGGGCC TTTTGCAGGC GGCAAAGACA CGGTTGTGAA
6961 TATTTTCCAC TTAAACACTA AAGCCGATGG CACGCTTAAA GCGGGAGGGT TTAAAGCTTC
7021 TCTTAGCACG AATGCGGCTC ATTTGAATAT CGGCGAAGGC GGTGTCAATC TGTCCAATCA
7081 AGCGAGCGGG CGCTCTCTTT TAGTGGAAAA CCTAACCGGG AATATCACCG TTGAGGGGAC
7141 TTTAAGAGTG AATAATCAAG TGGGCGGTGC TGCTGTGGCA GGCTCAAGCG CGAATTTTGA
7201 GTTTAAGGCT GGTGAAGACA CCAACAACGC CACAGCCACT TTTAATAACG ATATCCATCT
7261 AGGAAAAGCG GTGAATTTAA GAGTGGATGC TCATACAGCT TATTTTAATG GCAATATTTA
7321 TCTGGGAAAA TCCACGAATT TAAGAGTGAA TGGCCATAGC GCTCATTTTA AAAATATTGA
7381 TGCCAGTAAG AGCGATAACG GGCTAAACAC TAGCGCTTTG GATTTTAGCG GCGTTACAGA
7441 CAAAGTCAAT ATCAACAAGC TCACTACATC TGCCACTAAT GTGAACGTTA AAAACTTTGA
7501 CATTAAGGAA TTGGTGGTTA CAACCCGAGT TCAAAGTTTT GGGCAATACA CTATTTTTGG
7561 CGAAAATATA GGCGATAAGT CTCGCATTGG TGTCGTGAGT TTGCAAACGG GATATAGCCC
7621 GGCTTATTCT GGGGGCGTTA CTTTTAAAAG CGGTAAGAAA CTCGTTATAG ATGAAATTTA
7681 CCATGCCCCT TGGAATTATT TTGACGCTAG GAATGTTACC GATGTTGAAA TCAACAAAAG

(b)

B508A-T2A s1m2i2 scaffold_17 (s = 259 bp, m= 642bp, i = 432bp)

1 TGCTAAATTT AGCGTTTTGT GCGCTTAATT CTAAATTTTT ACTAAAATCG CTTATTGAGT
61 GGCTTCGCTC TAACGCTCTT TTTAGGTGAT ACTTTAAAGC CGCGCCTGCG GTGGCTTCGT
121 TTAAAGCGTA GCCGCTAAAC TAAAACTTTT TTTAAGAGCG CAGTTGTTGT AAGGCTTCTT
...
5461 TTTACAACAA AAAATCGCTT TGATGGACAC CCCACAAGGC ACGATTTGGG AGAAGCTTTT
5521 TTAAACGCCT CCAATTTTAC CTTTTTACAC GCTCTAGCCA CAAATTCTAG CAATATTGCT
5581 TTTTAATCTT GTTGAGTTTT ATGTTCATTT ACCTTAATTT GATAAAAATT GAATATTGGT
5641 TGTAGATACT ATATATTTAT AGCCTTAATC GTAAATGCAA CAGAAATTTT CTAGTCTAAA
5701 GTCGTACCCT TTGTGCAAAA ATCGTTTTAC AAGAAAAGAA GAAAGGAAAG AAATGGAAAT
5761 ACAACAAACA CACCGCAAAA TGAATCGCCC TTTAGTTTCT CTTGTTTTAG CAGGAGCGTT
5821 GATTAGCTCC ATACCGCAAG AGAGTCATGC TGCCTTTTTC ACAACCGTGA TCATTCCAGC
5881 CATTGTTGGG GGTATCGCTA CAGGCACCGC TGTAGGAACG GTATCAGGGC TTCTTAGCTG
5941 GGGACTCAAA CAAGCCGAAG AAGCGAATAA AACCCCAGAT AAACCCGATA AAGTTTGGCG
6001 CATTCAAGCA GGAAGAGGCT TTGATAATTT CCCCCACAAG CAATACGACT TATACAAATC
6061 CCTACTATCT AGCAAAATTG ATGGAGGTTG GGA<u>**C**</u>TGGGGG AATGCCGCTA GGCATTATTG
6121 GGTCAAAGAC GGGCAGTGGA ACAAACTTGA AGTGGATATG CAAAACGCTG TAGGGACTTA
6181 TAACCTTTCA GGCCTTATCA ACTTTACTGG TGGGGATTTG GACATCAATA TGCAAAAAGC
6241 CACTTTGCGT TTGGGCCAAT TCAATGGCAA TTCTTTCACA AGCTATAAGG ATAGCGCCGA
6301 TCGCACCACG AGGGTGAATT TTGACGCTAA AAATATCTTA ATTGATAATT TTGTAGAAAT
6361 CAACAATCGT GTGGGTTCTG GAGCCGGAAG AAAAGCCAGC TCTACGGTTT TGACCTTGAA
6421 AAGTTCAGAA AAAATTACAA GCCGTGAAAA CGCGGAAATC TCTCTTTATG ATGGCGCCAC
6481 GCTCAATTTG GTTTCAAGCT CAAATCAGAG CGTTGATCTA TATGGGAAAG TGTGGATGGG
6541 CCGTTTGCAA TACGTGGGAG CGTATTTAGC CCCTTCATAC AGCACAATAG ACACTTCAAA
6601 AGTGCAAGGG GAAATGAATT TTCGCCATCT CGCTGTGGGT GATCAAAACG CCGCTCAAGC
6661 GGGCATTATC GCTAATAAAA AGACTAATAT TGGCACACTG GATTTGTGGC AAAGTGCGGG
6721 GTTAAGCATC ATCACCCCTC CCGAAGGCGG TTATGAGAGT AAAACTAAAG ATAACCCTTC
6781 TCAAAACAAC CCTAAAAATG ACACGCAAAA AACAGAAATT CAACCCACGC AAGTCATTGA
6841 CGGGCCTTTT GCAGGCGGCA AAGACACGGT TGTGAATATT TTCCACTTAA ACACTAAAGC
6901 CGATGGCACG CTTAAAGCGG GAGGGTTTAA AGCTTCTCTT AGCACGAATG CGGCTCATTT
6961 GAATATCGGC GAAGGCGGTG TCAATCTGTC CAATCAAGCG AGCGGGCGCT CTCTTTTAGT
7021 GGAAAACCTA ACCGGGAATA TCACCGTTGA GGGGACTTTA AGAGTGAATA ATCAAGTGGG
7081 CGGTGCTGCT GTGGCAGGCT CAAGCGCGAA TTTTGAGTTT AAGGCTGGTG AAGACACCAA
7141 CAACGCCACA GCCACTTTTA ATAACGATAT CCATCTAGGA AAAGCGGTGA ATTTAAGAGT
7201 GGATGCTCAT ACAGCTTATT TTAATGGCAA TATTTATCTG GGAAAATCCA CGAATTTAAG
7261 AGTGAATGGC CATAGCGCTC ATTTTAAAAA TATTGATGCC AGTAAGAGCG ATAACGGGCT
7321 AAACACTAGC GCTTTGGATT TTAGCGGCGT TACAGACAAA GTCAATATCA ACAAGCTCAC
7381 TACATCTGCC ACTAATGTGA ACGTTAAAAA CTTTGACATT AAGGAATTGG TGGTTACAAC
7441 CCGAGTTCAA AGTTTTGGGC AATACACTAT TTTTGGCGAA AATATAGGCG ATAAGTCTCG
7501 CATTGGTGTC GTGAGTTTGC AAACGGGATA TAGCCCGGCT TATTCTGGGG GCGTTACTTT
7561 TAAAAGCGGT AAGAAACTCG TTATAGATGA AATTTACCAT GCCCCTTGGA ATTATTTTGA
7621 CGCTAGGAAT GTTACCGATG TTGAAATCAA CAAAAGAATT CTTTTTGGAG CCCCAGGAAA

(c)

B508A-T4 s2m2i2 scaffold_15 (s= 286 bp, m= 642 bp, i= 432 bp)

1 TTTTCGGCGT TGGCTTGTTC TAATGGGCTT AAATTGGTAG CGTTTTGTGG GGTTTCGTTT
61 AAATTTTCTG TATTTTTTAA TAAATCCTCT TGATCTATTA AATCTTTTTG TGTAGTATTC
121 TCTGTAGCAC CGCTATAGGA ACTCAACCTA TGGTTCACCT TTGCATCAGC ATTGAGTGTA
...
6481 TTAACTTAAT AAAAATTGAA CATTGGTTGT AGATACTGCA TATTTATAAC CTTAATCGTA
6541 AATGCAACAG AAATTTTCTA GTCTAAAGTC GCACCCTTTG TGCAAAAATC GTTTTACAAA
6601 AAGAAAGGAA AAAAATGGAA ATACAACAAA CACACCGCAA AATCAATCGC CCCATTATCT
6661 CTCTCGCTCT AGTGGGGGTG TTAATGGGCA CCGAACTAGG GGCTAATACG CCAAATGATC
6721 CCATACACAG CGAGAGTCGC GCCTTTTTTA CAACCGTGAT CATTCCAGCC ATTGTTGGGG
6781 GTATCGCTAC AGGCGCTGCT GTAGGAACGG TCTCAGGGCT TCTTAGCTGG GGGCTCAAAC
6841 AAGCCGAACA AGCCAATAAA GCCCCAGACA AACCCGATAA AGTTTGGCGC ATTCAAGCAG
6901 GAAGAGGCTT TGATAATTTC CCCCACAAGC AATACGACTT ATACAAATCC CTACTATCTA
6961 GCAAAATTGA TGGAGGTTGG GA<u>**C**</u>TGGGGGA ATGCCGCTAG GCATTATTGG GTCAAAGACG
7021 GGCAGTGGAA CAAACTTGAA GTGGATATGC AAAACGCTGT AGGGACTTAT AACCTTTCAG
7081 GCCTTATCAA CTTTACTGGT GGGGATTTGG ACATCAATAT GCAAAAAGCC ACTTTGCGTT
7141 TGGGCCAATT CAATGGCAAT TCTTTCACAA GCTATAAGGA TAGCGCCGAT CGCACCACGA
7201 GGGTGAATTT TGACGCTAAA AATATCTTAA TTGATAATTT TGTAGAAATC AACAATCGTG
7261 TGGGTTCTGG AGCCGGAAGA AAAGCCAGCT CTACGGTTTT GACCTTGAAA AGTTCAGAAA
7321 AAATTACAAG CCGTGAAAAC GCGGAAATCT CTCTTTATGA TGGCGCCACG CTCAATTTGG
7381 TTTCAAGCTC AAATCAGAGC GTTGATCTAT ATGGGAAAGT GTGGATGGGC CGTTTGCAAT
7441 ACGTGGGAGC GTATTTAGCC CCTTCATACA GCACAATAGA CACTTCAAAA GTGCAAGGGG
7501 AAATGAATTT TCGCCATCTC GCTGTGGGTG ATCAAAACGC CGCTCAAGCG GGCATTATCG
7561 CTAATAAAAA GACTAATATT GGCACACTGG ATTTGTGGCA AAGTGCGGGG TTAAGCATCA
7621 TCACCCCTCC CGAAGGCGGT TATGAGAGTA AAACTAAAGA TAACCCTTCT CAAAACAACC
7681 CTAAAAATGA CACGCAAAAA ACAGAAATTC AACCCACGCA AGTCATTGAC GGGCCTTTTG
7741 CAGGCGGCAA AGACACGGTT GTGAATATTT TCCACTTAAA CACTAAAGCC GATGGCACGC
7801 TTAAAGCGGG AGGGTTTAAA GCTTCTCTTA GCACGAATGC GGCTCATTTG AATATCGGCG
7861 AAGGCGGTGT CAATCTGTCC AATCAAGCGA GCGGGCGCTC TCTTTTAGTG GAAAACCTAA
7921 CCGGGAATAT CACCGTTGAG GGGACTTTAA GAGTGAATAA TCAAGTGGGC GGTGCTGCTG
7981 TGGCAGGCTC AAGCGCGAAT TTTGAGTTTA AGGCTGGTGA AGACACCAAC AACGCCACAG
8041 CCACTTTTAA TAACGATATC CATCTAGGAA AAGCGGTGAA TTTAAGAGTG GATGCTCATA
8101 CAGCTTATTT TAATGGCAAT ATTTATCTGG GAAAATCCAC GAATTTAAGA GTGAATGGCC
8161 ATAGCGCTCA TTTTAAAAAT ATTGATGCCA GTAAGAGCGA TAACGGGCTA AACACTAGCG
8221 CTTTGGATTT TAGCGGCGTT ACAGACAAAG TCAATATCAA CAAGCTCACT ACATCTGCCA
8281 CTAATGTGAA CGTTAAAAAC TTTGACATTA AGGAATTGGT GGTTACAACC CGAGTTCAAA
8341 GTTTTGGGCA ATACACTATT TTTGGCGAAA ATATAGGCGA TAAGTCTCGC ATTGGTGTCG
8401 TGAGTTTGCA AACGGGATAT AGCCCGGCTT ATTCTGGGGG CGTTACTTTT AAAAGCGGTA
8461 AGAAACTCGT TATAGATGAA ATTTACCATG CCCCTTGGAA TTATTTTGAC GCTAGGAATG

Figure S3. *vacA* genomic sequences extracted from the whole genomes of strains B508A-S1 **(a)**, B508A-T2A **(b)** and B508A-T4 **(c)**. Primers used for the detection of *s*, *i* and *m* regions are highlighted.

Table S1. Shared clusters between strains found using OrthoVenn2. **(a)** Strains B508A-T2A and B508A-T4. **(b)** Strains B508A-S1 and B508A-T2A. **(c)** Strains B508A-S1 and B508A-T4.

(a)

clusterID	Number of proteins	Swiss-Prot Hit
cluster 6	3	DUF874 family protein
cluster1409	2	hypothetical protein

(b)

clusterID	Number of proteins	Swiss-Prot Hit
cluster3	4	DUF874 family protein
cluster4	4	Outer membrane beta-barrel protein
cluster1405	2	Glycosiltransferase family 8 protein
cluster1406	2	hypothetical protein
cluster1407	2	Glycosiltransferase family 8 protein
cluster1408	2	DUF3519 domain-containing protein
cluster1410	2	DUF3519 domain-containing protein
cluster1411	2	Outer membrane protein family
cluster1412	2	Acetyl-coenzyme A synthetase
cluster1413	2	Alpha-1,2-fucosultransferase
cluster1414	2	Flagellar biosynthesis protein FlhF
cluster1415	2	Modification methylase HinfI

(c)

clusterID	Number of proteins	Swiss-Prot Hit
cluster5	4	DUF874 family protein
cluster1416	2	Modification methylase VspI
cluster1417	2	hypothetical protein
cluster1418	2	DUF697 domain-containing protein
cluster1419	2	Uncharacterized chromosomal cassette SCCmec type IVc protein CR006
cluster1420	2	hypothetical protein
cluster1421	2	outer membrane protein OipA
cluster1422	2	LPS heptosyltransferase family protein
cluster1423	2	Lipooligosaccharide biosynthesis protein lic2B
cluster1424	2	hypothetical protein
cluster1425	2	Flagellar biosynthesis protein FlhA
cluster1426	2	Type III restriction-modification system
cluster1427	2	hypothetical protein
cluster1428	2	ATP-binding protein
cluster1429	2	SAM-dependent methyltransferase
cluster1430	2	Uncharacterized protein HI_1472
cluster1431	2	Inner membrane ABC transporter permease protein YejE
cluster1432	2	Proline/betaine transporter
cluster1433	2	ATP-binding protein
cluster1434	2	cysteine desulfurase
cluster1435	2	Trigger factor

Table S2. Description of the unique genes found with Roary.

Unique genes	Description name
vacA	Vacuolating cytotoxin autotransporter (vacA_4)
flhA	Flagellar biosynthesis protein FlhA (*flhA_1*)
fucT_1	Alpha-(1,3)-fucosyltransferase FucT (*fucT_1*)
fucT_2	Alpha-(1,3)-fucosyltransferase FucT (*fucT_2*)
type-1 restriction enzyme	Putative type-1 restriction enzyme specificity protein MG438
hsdM	Type I restriction enzyme EcoKI M protein (*hsdM_2*)
rocC	Amino-acid permease RocC (*rocC_1*)
xerH	Tyrosine recombinase XerH (*xerH_3*)
hydB	Quinone-reactive Ni/Fe-hydrogenase large chain (*hydB*)
era	GTPase Era (*era_3*)
cpdA	3',5'-cyclic adenosine monophosphate phosphodiesterase CpdA
era	GTPase Era (*era_2*)
der	GTPase Der (*der_3*)
msrAB	Peptide methionine sulfoxide reductase *msrA/msrB* (*msrAB*)
yejE	Inner membrane ABC transporter permease protein YejE (*yejE_1*)
